# Study protocol of a randomized controlled trial of motivational interviewing-based intervention to improve adherence to continuous positive airway pressure in patients with obstructive sleep apnea syndrome: The MotivAir study

**DOI:** 10.3389/fpsyg.2022.947296

**Published:** 2022-08-25

**Authors:** Giada Rapelli, Giada Pietrabissa, Licia Angeli, Gian Mauro Manzoni, Ilaria Tovaglieri, Elisa Perger, Sergio Garbarino, Paolo Fanari, Carolina Lombardi, Gianluca Castelnuovo

**Affiliations:** ^1^Psychology Research Laboratory, Istituto Auxologico Italiano IRCCS, Milan, Italy; ^2^Department of Psychology, Catholic University of the Sacred Heart, Milan, Italy; ^3^Faculty of Psychology, eCampus University, Milan, Italy; ^4^Department of Pulmonary Rehabilitation, Istituto Auxologico Italiano IRCCS, Milan, Italy; ^5^Department of Cardiovascular, Neural and Metabolic Sciences, Sleep Disorders Center, Istituto Auxologico Italiano IRCCS, Milan, Italy; ^6^Department of Neuroscience, Rehabilitation, Ophthalmology, Genetics and Maternal-Infantile Sciences, University of Genoa, Genoa, Italy; ^7^Department of Medicine and Surgery, University of Milano-Bicocca, Monza, Italy

**Keywords:** sleep disorders, obstructive sleep apnea syndrome, continuous positive airway pressure, motivational interventions, adherence to CPAP, randomized controlled trial, randomized control trials (RCTs)

## Abstract

**Objective:**

This study aims to evaluate the effectiveness of the MotivAir program—a phone-based intervention based on Motivational Interviewing (MI) principles and techniques—in enhancing adherence to Continuous Positive Airway Pressure (CPAP) therapy among patients with Obstructive Sleep Apnea Syndrome (OSAS).

**Methods:**

A multicenter randomized controlled trial (RCT) design with random allocation at the level of the individual will be conducted to compare the impact of the experimental program (usual care *plus* MI) with a control group receiving usual care only in improving selected clinical and psychological parameters in the patients. A minimum sample of 80 participants (40 patients per group) will be recruited in each center according to the inclusion criteria. After the initial screening, participants will be randomly assigned to either the experimental group or the control condition. The program will last 180 days and will be delivered by a trained nurse. The impact of the MotivAir program on selected primary (adherence to CPAP in terms of average hours of usage per night and the Apnea-Hypopnea Index, AHI) and secondary (motivation, perceived competence, quality of life, sleepiness) outcomes will be measured at baseline, and after 1-, 3-, and 6-month from CPAP initiation.

**Discussion:**

Participants are expected to show an increased level of adherence to CPAP and to acquire the skills and self-confidence necessary to deal with the psychological consequences of their chronic condition.

## Introduction

Obstructive sleep apnea syndrome (OSAS) is a chronic illness characterized by a complete or partial obstruction of the upper airway. It has been demonstrated to be a risk factor for several diseases—including hypertension, impairment of cognitive functions, ischemic heart disease, and stroke (Garvey et al., 2015)—and to be correlated with other non-medical consequences—such as work-related injury, and motor vehicle crash (Garbarino et al., 2015, 2016)—that increase OSAS’s clinical and economic burden. Also, persons with OSAS often exhibit a poor health-related quality of life (HRQoL) (Lo Bue et al., 2020), which has a demonstrated negative impact on physical health outcomes (e.g., negative health perceptions, increased bodily pain, and poor physical functioning) and psychosocial functioning (e.g., mood disturbance, poor academic performance, and reduced social life) of individuals with OSAS (Timkova et al., 2020). In Italy, despite the impact of OSAS is highly underestimated due to substantial diagnosis gaps—it affects 27% of the adult population, of which 65% are males (Armeni et al., 2019).

Continuous positive airway pressure (CPAP) is the first-line treatment for moderate/severe OSAS. It increases the individuals’ clinical parameters and HRQoL by eliminating daytime sleepiness, and also decreases morbidity and mortality rates related to cardiovascular diseases (Luyster, 2017; Morsy et al., 2019; Lo Bue et al., 2020). Still, the effectiveness of the device is often limited by suboptimal adherence (Gay et al., 2006; Giles et al., 2006). Indeed, it has been suggested that improvements are more consistent with over 5.5 h of usage per night (Batool-Anwar et al., 2016; Bakker et al., 2019), but the literature reports that between 29 and 83% of patients enrolled in research studies use CPAP < 4 h per night (Bakker et al., 2016; Weaver, 2019) or refuse treatment (Baratta et al., 2018; Rezaie et al., 2021).

Adherence to CPAP treatment depends on several factors, including individual characteristics, features associated with the device (Crawford et al., 2014; Batool-Anwar et al., 2016) and its side effects, as well as psychological and social determinants (Crawford et al., 2014; Sanna and Lacedonia, 2018).

Understanding barriers and facilitators to CPAP adherence is vital for the development of effective interventions. Over the years, it has become evident that suboptimal adherence is largely predicted by psychological measures of behavior change—including motivation and perceived self-efficacy (Philip et al., 2018; Wong et al., 2020; D’Rozario et al., 2021). For example, in a multiethnic sample of 248 patients, self-efficacy (which describes the extent to which a patient believes that he or she is capable of attaining positive outcomes from treatment) was significantly associated with adherence to CPAP after adjusting for several other potential determinants (Wallace et al., 2013).

Motivational Interviewing (MI) is often used in healthcare settings to increase motivation to change and self-efficacy in patients with chronic illnesses (Pietrabissa et al., 2012, 2013, 2015; Pietrabissa, 2018; Nedjat-Haiem et al., 2019; Scott et al., 2019), and has a demonstrated positive impact in increasing adherence to CPAP among patients with OSAS (Miller and Rollnick, 2002, 2013; Rapelli et al., 2021). Consistent with several models of behavior change, including the transtheoretical model of change (TMC, Prochaska and DiClemente, 1983), this approach is mainly aimed at addressing the individuals’ inner ambivalence to change. Expressing empathy, developing discrepancy, rolling with resistance, and supporting self-efficacy are the principles encompassed by MI on which intervention strategies with existing empirical support (Olsen et al., 2012) are based to help patients to develop specific goals and increase their readiness to change.

A few studies have considered adherence to CPAP as compliance maintained over time correlated with disease awareness and change to a healthier lifestyle (e.g., Weaver and Sawyer, 2010) but to our knowledge, even fewer are studies that address these aspects in correlation with technological advances such as telemonitoring and telephone assistance (e.g., Hu et al., 2021). However, the recent study conducted by Hwang et al. (2018) shows that telemonitoring with automated feedback messages improved adherence to CPAP at 90 days in OSAS patients. Furthermore, beyond the single clinical and sociodemographic factors, the studies of Aloia et al. (2007) and Budhiraja et al. (2007) indicate that long-term adherence is determined by the results conquered in the first 2 weeks of treatment. In fact, telemedicine interventions and action plans provided at home are increasingly supporting patients in health communication, self-monitoring, and self-treatment. These interventions have made information easily accessible, helping patients to early detect symptoms, with more timely treatment, reduction in hospitalizations, and improved health-related quality of life (Hu et al., 2021). Furthermore, telemedicine-based interventions may be able to facilitate assisting patients with physical limitations and those who need a frequent evaluation of their health status. They also have the potential to foster greater patient engagement and less costly interventions (Hu et al., 2021). Although more research into the effectiveness of these telemedicine interventions is still needed because research in patients with OSAS is underdeveloped.

More investigations should focus on the use of the different formats of delivering motivational interventions including the use of new technologies, in fact to our knowledge results regarding the effects of remote-MI interventions for naïve patients using CPAP are still scant and inconsistent. Furthermore, only one RCT (Sparrow et al., 2010) tested the effect of a MI-based intervention using telemedicine in the long-term after 12 months from CPAP initiation, and collected interesting results. In fact, research that can effectively be provided digitally to patients with OSAS using CPAP could represent advantages and reduced costs (Appel et al., 2011; Bus et al., 2018).

Concerning the educational and formative aspects, Olsen et al. (2012) applied an educational program to naive patients that integrated the MI—which was recognized as best practice by the Agenzia di Tutela della Salute of Lombardy in Italy for the promotion of a healthy lifestyle—based on the work of Rollnick and Miller (1995) with the modifications indicated by Aloia et al. (2004) for OSAS patients.

For these reasons, we develop the MotivAir project—a phone-based motivational intervention that integrates telemedicine and telephone assistance based on MI principles and techniques to support adherence to CPAP in naïve patients with OSAS. This is the first study in Italy that will use MI-based intervention for OSAS patients by integrating telemedicine and telephone assistance and in-person sessions. Furthermore, it will be based on personalized care plans in response to the patient’s profile (e.g., patient age, socio-demographic variables, and distance to hospital) and this approach could identify who needs intensive care and further increase CPAP adherence. A personalized-based intervention represents a golden standard for the cost-effective impact, but only one study has been achieved with personalization of care for patients with OSAS using CPAP.

Rudilla et al. (2021b) already showed increased compliance with the device, as well as increased self-efficacy, and quality of life in terms of daily activities, and social relationships in CPAP-naive patients with severe OSAS after receiving a personalized motivational intervention plan. The purpose of this randomized controlled trial (RCT) will be to determine whether MI-based strategies delivered by a nurse, and added to a standard pulmonary rehabilitation program, will be superior to usual care in improving adherence to CPAP. Specifically, we hypothesize that adding MI feedback to standard care will result in improved adherence to CPAP in terms of average hours of usage per night and increased Apnea-Hypopnea Index (AHI) after 90 days from CPAP initiation. We also hypothesize that results will be maintained or increase at 6 months of follow-up and that patients will also show increased levels of motivation, perceived competence, and sleepiness.

## Methods/design

The effectiveness of the MotivAir program will be assessed in a multi-center randomized controlled study with two arms: an experimental arm with TAU plus personalized MI carried out by a trained nurse (MotivAir group), and a TAU control.

The study was approved by the Ethical Committee of the Istituto Auxologico Italiano, Italy (ID: 2021_03_23_02). All procedures performed in the study will be run following the ethical standards of the institutional and/or national research committee and with the Helsinki Declaration and its later amendments or comparable ethical standards.

## Study population

All patients diagnosed with OSAS and CPAP referring to the IRCSS Istituto Auxologico Italiano, San Giuseppe hospital, and San Luca Hospital will be screened for admission into the study during their first week of a pulmonary rehabilitation program. Patients will be eligible if meeting the following inclusion criteria: (1) being over 18 years old; (2) have a diagnosis of OSAS confirmed by polysomnography; (3) being recommended for treatment with CPAP; (4) being naïve to this type of intervention; (5) being fluent in the Italian Language; and (6) having signed the written informed consent to participate in the study. Exclusion criteria will be: (1) use of oxygen therapy > 2 l/min; (2) history of severe cognitive disorders; (3) history of COPD: FEV1/FVC (Tiffeneau Index) ≤ 60% with FEV1 ≤ 50%; (4) dyspnea on exertion (Borg > 6); (5) diagnosis of Long COVID or COVID-19 infection < 4 months; (6) chronic heart failure (NYHA: Grade III and IV); (7) unstable ischemic heart disease; 8) presence of visual, or hearing impairments that will prevent the patients from following the intervention and filling in the questionnaires.

Before enrolling in the study, all patients will be informed in detail about the study criteria and procedure and will be asked to sign the consent form to participate.

## Randomization procedure

**FIGURE 1:**
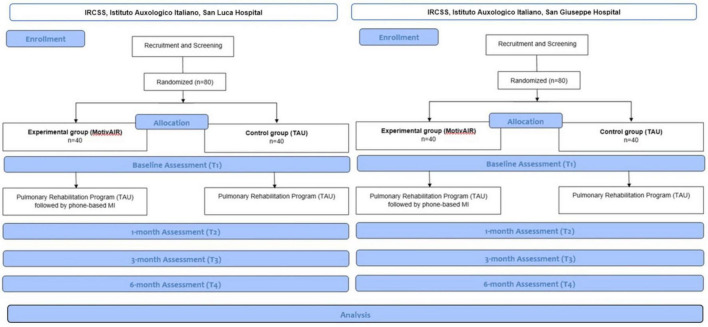
Flow chart of the MotivAIR study.

Randomization will be stratified within each center through permuted randomized blocks. Randomization will take place after the baseline measurements (Figure 1).

## Sample size calculation

A sample of 80 patients (40 in each group) will allow detection as a significant effect size of 0.67 (based on the average daily difference in apnea hours between groups) measured at the 6-month follow-up, a first type error of 0.05, a power of 80% and a drop-out of 10%. Data related to the effect size were retrieved from the PIMA study (Rudilla et al., 2021b), while the mean daily difference in apnea hours and the standard deviation were provided by the researchers. The sample size was assessed using PASS 14 software (Power Analysis and Sample Size Software (2015). NCSS, LLC. Kaysville, Utah, United States, ncss.com/software/pass).

### Measures

*Demographic information* about age, gender, education, and civil status will be self-reported at baseline.

*Clinical variables* will be recorded during the initial visit with the physician and retrieved by the patients’ medical records by a clinical psychologist working in the clinic, independent from the study. They will include the (1) *Epworth Sleepiness Scale* (ESS; Johns, 1991; Vignatelli et al., 2003) as a measure of daytime sleepiness. Respondents will be asked to rate, on a 4-point scale (0–3), their usual chances of dozing off or falling asleep while engaged in eight different activities. The ESS score can range from 0 to 24. The higher the ESS score, the higher that person’s average daytime sleepiness; and the (2) *Apnoea-Hypopnea Index (AHI)*, which determines the presence and severity of OSAS.

### Primary outcome

The primary outcomes of this study will be the participants’ *adherence to CPAP therapy* (mean hours/night) and the AHI score after 180 days from the treatment beginning. CPAP usage (hours/night) will be automatedly recorded by the device, while AHI will be measured by polysomnography during follow-up visits at the hospital.

Data will be also analyzed after 30 and 90 days of treatment as a secondary outcome.

### Secondary outcomes

The following psychological measures will be collected at baseline (T1), and after 1 month (T2), 3 months (T3), and 6 months (T4) from treatment beginning.

Questionnaires will be administered by a clinical psychologist working in the clinic and independent from the study.

The *Questionnaire of Evaluation of Perceived Competence in Adherence to CPAP in OSAS* (CEPCA; Rudilla et al., 2021a) will be used to assess the participants’ perceived self-efficacy. It comprises 13 items grouped into three categories: knowledge of OSAS and its associated risk; expectations regarding CPAP treatment; and confidence in overcoming barriers associated with the use of the device. The scores obtained in the CEPCA are positively related to the quality of life and motivation and negatively related to daytime sleepiness.

The participants’ perceived quality of life will be assessed using the *Visual Analogical Well-being Scale for apnea* (Masa et al., 2011).

Moreover, the individuals’ motivation will be assessed according to the Prochaska and Di Clemente transtheoretical model of change Prochaska and DiClemente (1982). Based on the answer to a single question [“What is the level of motivation that motivates you to undertake CPAP therapy? This includes a 5-point Likert response scale (none, a little, somewhat, motivated, very motivated)?”] each patient will be classified as in the: pre-contemplation (no motivation), contemplation (low motivation), determination (some motivation), action (quite motivated), or maintenance (high motivation) motivational stage.

## Procedure

Socio-demographic (age, level of studies, presence of caregiver at home), psychological (motivation and perceived competence) clinical variables (somnolence and apnea-hypopnea index), and variables concerning the time the patient spends from home to the care center and the confidence in using electronic applications will be used to classify patients’ adherence to treatment as low, moderate or high, and to create a tailored motivational treatment plan for the subjects assigned to the experimental group. Patients showing low adherence will receive a more intensive care plan, than those recognized as highly compliant with CPAP use. The results are patients with the “a” profile (characterized by autonomy and mobility, predisposition to remote-controlled follow-up), patients with the “b” profile (need for more intensity in follow-up), or patients with the “c” profile (more difficulties to move around and require more intensive treatment). Profile “d” was applied to patients who were professional drivers, as they require specific interventions based on their occupation (see Rudilla et al., 2021b).

Participants assigned to the control group will receive a usual pulmonary rehabilitation program for patients with OSAS receiving CPAP therapy, which is a standard technical training comprising information regarding the use, maintenance and safety measures of the device, plus a home inspection delivered by a technician who has the only task of doing maintenance to the machinery.

In addition, subjects in the MotivAir group will follow a telephone-based intervention—lasting ~45 min—based on MI principles and techniques delivered by a nurse. For the specific purpose of the study, the nurse will preliminarily receive 8 h of MI training provided by two psychologists experts in the MI approach (authors GR and GP). During the training, the nurse will learn about the collaborative, evocative, and client-centered spirit of MI, and the characteristics that define each stage of change and will be instructed to apply the basic communication and listening skills of this approach (open-ended questions, affirmations, reflective listening, summarizing). Then she will learn to reinforce and elicit change talk, while also responding in ways that reduce counter-change talk and to roll with the patients’ inner resistance to change by affirming the patients’ autonomy and reflecting their resistant speech with empathy. Next, the issues of how and when to introduce the development of a change plan, and enhance the individuals’ commitment to change will be addressed.

The treatment plan will be implemented based on each patient’s level of adherence. To low adherence (score below 16 on the ESS and CEPCA questionnaires) a more intensive telephone-based assistance (days 1, 8, 16, 30, 90, 120, 180 from the end of the rehabilitation period) will follow. In the case of high adherence (score greater than or equal to 16 on the ESS and CEPCA questionnaires), phone encounters will be set on days 1, 8, 30, 90, and 180 after treatment termination.

At 1-, 3- and 6-month follow-up, adherence to the device will be assessed by automatedly recorded CPAP usage (hours/night) and AHI. Changes in the selected psychological variable will be also examined.

## Treatment fidelity

MI sessions were audio-recorded, transcript verbatim, and—some of them—randomly selected to be critically supervised by an expert in the field not involved in the study (Martino et al., 2008).

## Statistical analysis

Descriptive statistics (means ± SD, or median and interquartile ranges, as appropriate) will be used to describe the study sample with regard to baseline characteristics. Before selecting the most appropriate statistical tests, assumptions for parametric analyses will be checked. Continuous variables will be reported as mean and standard deviation (or median and interquartile range when needed). Categorical variables will be reported as absolute and relative frequency. The mean daily apnea hour will be compared between groups by repeated-measures analysis of variance. To account for the correlation of within-patient measurements, a linear mixed model with a covariance variance matrix chosen based on the lowest Akaike Information Criterium (AIC) index value will be used. The model will also possibly include some characteristics of patients that are found to influence the level of expected adherence identified with preliminary multinomial logistic regressions. All tests will be two-tailed and the *p*-value will be significant if less than 0.05. Analyses will be conducted with SPSS statistical software.

## Expected results

The results of this multicentric RCT will provide evidence for the effectiveness of the MotivAir program in supporting adherence to CPAP use among patients with OSAS.

Specifically, based on the results of previous research (e.g., Sparrow et al., 2010; Aloia et al., 2013; Dantas et al., 2015; see for a review: Rapelli et al., 2021), participants assigned to the experimental group are expected to show increased clinical and psychological parameters compared to the control condition. Indeed, according to MI theoretical assumptions, positive changes in the post-treatment levels of readiness to change and perceived self-efficacy will result in increased adherence to behavioral change plans.

Furthermore, it is expected that personalized MI treatment based on the individuals’ initial levels of adherence to CPAP therapy will result in further improved outcomes up to 180 days after completion of the standard rehabilitation program compared to TAU.

Finding from this study will, therefore, contribute to the evidence-based knowledge of how MI may enhance the cost-effectiveness of clinical interventions to increase adherence to CPAP among patients with OSAS and will provide suggestions on how to implement traditional health care services.

## Author contributions

GR, GP, and GC conceived the study, participated in its design and coordination, and helped to draft the manuscript. LA and GM participated in the study design and made substantial contribution to the manuscript drafting. EP, PF, and CL participated in the study design and revised the draft critically. IT and SG participated in the study design and helped to draft the manuscript. All authors read and approved the final manuscript.

## Abbreviations

AHI: Apnea-Hypopnea Index
COPD: Chronic Obstructive Pulmonary Disease
CPAP: Continuous Positive Airway Pressure
ESS: Epworth Sleepiness Scale
FEV1: Forced Expiratory Volume in the 1st second
FVC: Forced Vital Capacity
MI: Motivational Interviewing
NYHA: New York Heart Association.
