# Corrigendum: Study protocol of a randomized controlled trial of motivational interviewing-based intervention to improve adherence to continuous positive airway pressure in patients with obstructive sleep apnea syndrome: The MotivAir study

**DOI:** 10.3389/fpsyg.2023.1156627

**Published:** 2023-02-15

**Authors:** Giada Rapelli, Giada Pietrabissa, Licia Angeli, Gian Mauro Manzoni, Ilaria Tovaglieri, Elisa Perger, Sergio Garbarino, Paolo Fanari, Carolina Lombardi, Gianluca Castelnuovo

**Affiliations:** ^1^Psychology Research Laboratory, Istituto Auxologico Italiano IRCCS, Milan, Italy; ^2^Department of Psychology, Catholic University of the Sacred Heart, Milan, Italy; ^3^Faculty of Psychology, eCampus University, Milan, Italy; ^4^Department of Pulmonary Rehabilitation, Istituto Auxologico Italiano IRCCS, Milan, Italy; ^5^Department of Cardiovascular, Neural and Metabolic Sciences, Sleep Disorders Center, Istituto Auxologico Italiano IRCCS, Milan, Italy; ^6^Department of Neuroscience, Rehabilitation, Ophthalmology, Genetics and Maternal-Infantile Sciences, University of Genoa, Genoa, Italy; ^7^Department of Medicine and Surgery, University of Milano-Bicocca, Monza, Italy

**Keywords:** sleep disorders, obstructive sleep apnea syndrome, continuous positive airway pressure, motivational interventions, adherence to CPAP, randomized controlled trial, randomized control trials (RCTs)

In the published article, there was an error in affiliation for Giada Rapelli. Instead of “^1^Department of Psychology, Catholic University of the Sacred Heart, Milan, Italy, ^2^Psychology Research Laboratory, Istituto Auxologico Italiano IRCCS, Milan, Italy,” it should be “^1^Psychology Research Laboratory, Istituto Auxologico Italiano IRCCS, Milan, Italy.”

In the published article, there was an error in affiliation for Elisa Perger. Instead of “^5^Department of Cardiovascular, Neural and Metabolic Sciences, Sleep Disorders Center, Istituto Auxologico Italiano IRCCS, Milan, Italy, ^6^Department of Medicine and Surgery, University of Milano-Bicocca, Monza, Italy,” it should be “^5^Department of Cardiovascular, Neural and Metabolic Sciences, Sleep Disorders Center, Istituto Auxologico Italiano IRCCS, Milan, Italy.”

In the published article, there was an error in affiliation for Gian Mauro Manzoni. Instead of “^2^Psychology Research Laboratory, Istituto Auxologico Italiano IRCCS, Milan, Italy, ^3^Faculty of Psychology, eCampus University, Milan, Italy,” it should be “^3^Faculty of Psychology, eCampus University, Milan, Italy.”

The authors apologize for these errors and state that this does not change the scientific conclusions of the article in any way. The original article has been updated.

